# The Lived Experiences of Deep Brain Stimulation in Parkinson's Disease: An Interpretative Phenomenological Analysis

**DOI:** 10.1155/2019/1937235

**Published:** 2019-02-03

**Authors:** Suzette Shahmoon, Jonathan A. Smith, Marjan Jahanshahi

**Affiliations:** ^1^UCL Institute of Neurology, Sobell Department of Motor Neuroscience and Movement Disorders, Queen Square, London, UK; ^2^Department of Psychological Sciences, Birkbeck University of London, London, UK; ^3^The Clinical Hospital of Chengdu Brain Science Institute, MOE Key Lab for Neuroinformation, University of Electronic Science and Technology of China, Chengdu, China

## Abstract

Deep brain stimulation of the subthalamic nucleus (STN-DBS) is an effective treatment for Parkinson's disease (PD). In this study, we used an interpretative phenomenological analysis to explore how 10 male people with PD experienced life after STN-DBS surgery. Two themes emerged. The first, “Healed and relieved: all that glitters is not gold,” highlights the benefits and the personal “costs” of surgery. The second, “The change within: new interpretations of the present and future unfold,” explores how patients reinterpreted their lives as individuals and members of society in the present and as they face their future. Relief, gratitude, disappointment, and the need for social support are expressed as well as a new appraisal of values and the future. STN-DBS alters the life course of people with PD, and this study provides new insight into psychological and social issues that surgery raises for the patient and their family system. These psychosocial issues should be taken into account when preparing the patient and their family for surgery or supporting them postoperatively.

## 1. Introduction

Parkinson's disease (PD) is a progressive, chronic disorder characterized by tremor, rigidity, muscle stiffness, and gait problems [1]. When symptoms of PD become harder to treat with medication and medication-induced side effects emerge, deep brain stimulation (DBS) of the subthalamic nucleus (STN) is an effective treatment for the motor symptoms of moderate/severe PD [2–4].

The aim of surgery is to improve the quality of life (QoL) of people with PD by improving the motor symptoms [5]. Therefore, current STN-DBS literature focuses heavily on outcome measures for improvement of the motor symptoms such as the Unified Parkinson's Disease Rating Scale and quality of life such as the PDQ39 [6]. These scales have been useful in quantifying the positive changes heralded by surgery but are not sensitive enough to show how these remarkable improvements affect the lived experience of the person with PD. They do not show how the changes produced by DBS impacts on experiential and inner life of the patients, which can be evaluated through qualitative research. Qualitative studies can inform professionals on how to better prepare individuals and their families for surgery and how to support them better after the operation.

To date, only a few qualitative studies have assessed the lived experience of people with PD after DBS surgery. Most of the studies have focused on psychosocial adjustment (for review, see [7, 8]), the experience of surgery [9], or the relief surgery brings [10, 11].

In these studies, the patients interviewed have commented on how DBS has changed the way they feel about themselves, with some feeling dehumanized by the implanted electrodes and stimulating device [12–14]. Others found DBS had removed their impetus to live, as they no longer had a disease to fight [15, 16]. Marital breakdown occured as a result of surgery due to spouses feeling liberated from their caring duties and from patients not wanting to resume roles such as working or sharing household duties, when their spouses expected them to take on new responsibilities with their improved motor function [16, 17].

The more positive views of DBS spoke of symptomatic relief (for review, see [7]). Others regarded their implants as a visible confirmation, almost a badge of honour of their illness [10]. One study focused on occupational ability and the effects of DBS on PD before and after surgery as measures of success [18].

What is evident from all these previous studies is that patients need to reinterpret who they are and their place in the world after DBS surgery. The rapid change to sudden health can affect the way the patients and their family act and interact with each other and within the world, and the “merging” with technology can influence self-image. With this in mind, the aim of this study was to explore how PD patients reinterpret their life using interpretive phenomenological analysis (IPA). IPA is a method of collecting and analysing information pertaining to how one makes sense of one's life-world [19]. Unlike previous studies, this study is not designed to investigate postsurgical psychosocial adjustment. Instead, it aims to make a detailed analysis of what living with STN-DBS means to the person with PD [20] as an individual and a member of the society. We therefore interviewed ten male PD patients, under the age of 70, who were all married or in a committed relationship when first diagnosed with Parkinson's disease, and have had DBS of the STN at least for nine months. The aim was to establish the effects surgery has had on their life by asking open-ended questions about their daily living, their relationships with others, and their views of themselves.

## 2. Methods

### 2.1. Participants

This study used an interpretative phenomenological analysis and hence only requires a small homogenous sample [19]. Therefore, 10 male people with PD under the age of 67 years (age range 46–67) who were or had been married and who had STN-DBS surgery up to nine months prior to interview were recruited from among the operated patients and interviewed at the Functional Neurosurgery Unit at the Hospital of Neurology and Neurosurgery in London. The demographic and clinical details of the participants are presented in Table 1.

### 2.2. Procedure

Ethics approval was granted by the NHS Bloomsbury research ethics committee and Birkbeck University ethics committee. Informed consent was obtained from all participants. All names have been changed to protect confidentiality.

A semistructured interview schedule was devised, aimed at understanding how DBS surgery has impacted on their lived experiences. Interviews ranging between 30 and 60 min were conducted. Interviews were recorded and transcribed verbatim.

Each interview was analyzed in isolation of the others so as to maintain as much objectivity and open-mindedness with regards to emergent themes. One interview was discounted due to the participant's severe speech issues and problems with communication.

Analysis was conducted following the guidelines set out by Smith, Flowers, and Larkin [19]. Upon the initial reading of the text, there was an annotating phase of analysis. Descriptive, linguistic, and conceptual comments were noted followed by identification of emergent themes within the text. Similar themes repeated throughout the text were then consolidated and given an appropriate theme title.

The emergent themes were then clustered into superordinate themes. This process was undertaken for each participant's interview. Once all ten interviews were analyzed in this way, they were compared looking for the convergence and divergence captured within each participant's lived experience.

## 3. Results

The following main themes emerged from the 10 interviews: “A rebirth after DBS,” “Facing reality and disappointment,” “The social support buffer,” “To control or be controlled,” “To help or be helped,” “Life is reevaluated,” and “The future is less predictable.” These were clustered into the following two superordinate themes:

1. Healed and relieved: all that glitters is not gold
2. The change within: new interpretations of the present and future unfold

### 3.1. Healed and Relieved: All That Glitters Is Not Gold

#### 3.1.1. A Rebirth after DBS

DBS has the ability to control the motor symptoms and give a sense of turning back the clock for the PD patient; a sentiment reflected in this theme.

Dean: *I just feel much more happy in myself […] I know I'm back to me again*.

We see here Dean reconnects to his sense of self, thanks to the alleviation of his PD symptoms. This statement suggests PD previously challenged him at an identity level. Surgery has restored his self-perception allowing him to “know” himself once more.

Most of the men interviewed described how being free from PD symptoms made them feel like themselves again. For Henry, this meant having more mental clarity:

*I just got a bit more clarity and, you know, I just went back to the meaning of normal*.

The word normal here expresses how his experience of PD made him feel less than normal and how surgery has facilitated this return to normality.

This sense of being renewed and reborn is not just felt by the patient but also seen through the lens of loved ones. Barry talks about this global recognition of rebirth.

For Barry, the effect of DBS is even more fundamentally satisfying:

It's definitely improved my ability to function as a human being, to function as an independent member of society.

This statement suggests that PD had an almost dehumanising effect on him that DBS has managed to restore. DBS has been the conduit enabling him to feel like a functioning human and return to society as if it was a social club he was excluded from due to his illness.

DBS helps the patient to reconnect to their true sense of self as an individual and as a member of society. However, normality is subjective, and DBS is not a cure. DBS masks the symptoms of PD well, but how long lasting that masking is, is subjective.

#### 3.1.2. Facing Reality and Disappointment

The results of surgery, for some, either did not last as long as desired or did not fix as much as it was hoped, causing disappointment that expectations were not met.

For as much as Barry felt like an “*independent member of society*,” after surgery, he still reported having ON's and OFF's and “*any swing during the day is a swing and I'm still having to cope with it every day. So in a sense that's kind of disappointing.*”

There is a duality in the tone of Barry's account, whereas previously, there was sheer delight at his being free to function in society; Barry points out that PD is disappointingly still very present in his life. At least half of the men interviewed shared this experience. Barry continues:

*There's still the ON/OFF […] There is still pain*.

He expresses some frustration at still having to regulate his physical state even though he has the added benefit of the remote control. Later, in the interview, he explains he does not “bother” to turn down his stimulation to reduce his dyskinesia suggesting that he has surrendered to his symptoms. In this case, DBS has not motivated Barry to manage his symptoms rather it has reinforced the reality that PD is still present and he is not in control of his motor state.

Unlike Barry, Charles no longer worries about his old symptoms but is now plagued by new ones. Charles' symptoms were well managed by his medication but at the cost of emotional, cognitive, and psychological stability. Charles has now had another trade-off situation where his improved motor functionality has been at the cost of impaired communication: speech is slurred and laboured and his ability to write is almost gone. Even though he is grateful for what DBS has accomplished, he laments:

I do wish […] there was some way I could take some part of it back […] if anything's come up that's gonna get me speech right I'd have the operation again

Wanting to exchange some of his new symptoms for the old suggests disappointment with the results of surgery. Charles finds it hard to accept that he cannot communicate freely. The admission that he is even willing to repeat the surgery shows to what extent he is desperate to regain what he has lost.

Gary, the most recently interviewed of the group, underwent reimplantation not long after his interview. The effects of DBS were very short lived for Gary. “*After about two months we were starting to see some negatives creep in.*”

One year since Gary's surgery, the positive effects of DBS are barely visible any more. He has an almost fatalistic view of this when he says, “*If this is as good as it gets then we'll live with this.*”

This sums up the general sentiment of the whole group. There was a general feeling of gratitude and acceptance that any relief was better than no relief irrespective of expectations not being met; however, the underlying sentiment of disappointment remained.

We note here Gary's use of the pronoun “we,” emphasizing the fact that DBS does not only affect him but his wife too. PD affects both patient and carer, and how the changes associated with DBS are perceived and how the patient is supported can affect how well they adjust to life after DBS surgery.

#### 3.1.3. The Social Support Buffer

Half of the patients interviewed alluded to the importance of good social support. For some, social support was very present prior to surgery but diminished after DBS.

Gary's PD journey has been a joint one for him and his wife. It is a collaborative effort. There is a shared duty of care between the couple, and this extends into their local community.

*We had a situation where my wife was not well at all at a period where I was not well and we had friends deliver […] meals, three times a day for six weeks*.

However, after surgery,

*a lot have felt that PD has gone and I don't hold that against them at all…but no I think the support is there. Encouragement all the times. That's been one of the major contributions to the way we've handled that, the PD*.

The words “I don't hold that against them” suggests a measure of disappointment in the current level of support being given. However, Gary seems to come to a realisation while speaking that he still receives support but it has evolved. DBS causes such dramatic, visible changes that friends and family come to believe that PD is no longer an issue for the patient, allowing them to withdraw support. While reflecting back, Gary becomes aware that he is supported emotionally rather than physically.

Henry's experience of PD, however, has been the polar opposite of Gary's. When Gary reflects on the support he has received from his wife, Henry denies any evidence of her supporting him and states,

I know my wife really suffered […]. I cope with things a lot better than she does.

Throughout his interview Henry reflects on his independent nature, his ability to cope, and his wife's lack of coping and supporting skills. DBS has allowed him the clarity to understand that his wife's lack of support was not personal but a reflection of her struggle to cope with his PD.

DBS gave Henry the ability to understand the tension in his marriage from a different perspective. It is only now that he is able to understand that the two states of coping and supporting can rely heavily on each other.

DBS helped afterwards, you know I could see the clarity.

It is hard to know if Henry really has had as little support as he thinks he has or if he was blind to it in his effort to maintain his independence.

I think perhaps that in hindsight, having the good core of friends and family is very, very important. The support network was something probably that I didn't have.

While reflecting, he realizes the importance of allowing himself to be supported and having good social support because it

Probably would have made things more comfortable for me.

There is a common understanding that being supported by friends and family is important. DBS surgery can cause dramatic change and those changes affect everyone in the family system, meaning they must also adjust accordingly. It would seem the more stable social support remains, after DBS surgery, the more likely postsurgical adjustment will be smooth.

### 3.2. The Change within: New Interpretations of the Present and Future Unfold

#### 3.2.1. To Control or Be Controlled

PD depletes the patient of their sense of agency. Daily activities are exchanged for pill-taking routines and management of side effects. DBS can reverse this almost immediately, and the introduction of a remote control can help find even more control over Parkinsonian symptoms as they occur throughout the day.

All of the patients interviewed were given their own remote control but not all used it. Most had shown some level of confusion around either how to use their controller or how often they could/should use the device. The more newly operated patients tended to abstain from use as much as possible, and a few such as James, handed over responsibility to their wives.

James: *My wife uses it on me. I haven't got used to using it yet. But we have upped it ourselves. My wife said to me yesterday, “Should we up it now.” No. No. I'm alright.*

Those patients who did use the remote control more frequently tended to be the ones who were more in need and hence became more accustomed to independent use over time. Adam is quite comfortable using his remote control and has found a way of managing his symptoms quite meticulously,

I devised this method of up and down because I can pinpoint exactly when the Levodopa is working and when it is not

Adam has struggled with impulse control disorders due to medication. Having control over the amount of stimulation he gets means he now has much more stability during the day, and this has had a positive impact on his family.

because I'm on a more even keel.

The remote control has brought an element of peace and understanding within his family system. His family has a stronger sense of security and understanding of who he is again.

Barry's experience of having a remote control is very similar to Adam's; however, unlike Adam, when Barry describes his need for the remote, it comes across as a burden.

I need to watch how I'm feeling inside, I need to watch when I'm going to take the next pill. Am I feeling ok? Am I coming OFF? You know, shall I take a pill? That's a constant thought process that goes on in my head the whole time and one of the things I can do now is I can use the remote control

Whereas earlier, Barry only needed to concern himself with when to take his next pill, but now he must also control his remote control usage. He states

I do not bother switching them (dyskinesias) off because this thing would be in my hand 24/7 […] it would just be too much to think about.

For as much as DBS has helped to control his symptoms, it seems that Barry struggles with the urge to control his DBS.

#### 3.2.2. To Help and Be Helped: Nothing Is Taken for Granted Any More

PD remains very much in the background making the patient reassess their relationship to their illness, the management of their illness, and the support of those around them. Around half of the interviewees recognised and paid tribute to the help they had received. For many, this sparked a new sense of benevolence, and for most it was their impetus to take part in this study.

*That's why I'm trying to help people like you. […] the fact that the hospital helped me so much, it makes such a big difference to me that I really want to repay it back*.

James feels indebted to the surgical team because DBS has been life changing. He volunteered to be interviewed in the hope that it would not only further research in the field but also help the hospital that changed his life.

Ed also finds he likes to give his time to people with PD now that he has had DBS surgery. Ed is a musician whose PD made it virtually impossible for him to play music. After surgery, he is playing music again.

I'm getting paid again, well I donate everything that I earn to an orphanage.

Ed is so thrilled he has the opportunity to play music again, that he wants others to also benefit from the success of his surgery.

Whether it is financial aid, time, or hope, DBS seems to trigger in some the desire to help others.

#### 3.2.3. Life Is Reevaluated

DBS gives the patient the freedom to stop focusing on their physical state and focus on living.

Adam: *It's made me not want to give it up. The second bite of the cherry. That's why I'm trying to keep as fit as I can….I don't want to go backwards.*

The initial theme of rebirth is revisited here and expanded upon as the PD patient now has a unique perspective on living. There is a new appreciation for independence and life in general. Adam feels reborn knowing how good life can be and how quickly that can be taken away. He is now placing importance on his physical state staying fit in the hope that it will slow the progression of the disease and stave off the symptoms for as long as possible. This sentiment is repeated by Ed.

Ed: *What's important now is totally different to what it used to be. Re-evaluation I put it down to, I think, re-evaluate what's important in life.*

This encapsulates the sentiment felt by most of the DBS patients. Having lived for years at the mercy of Parkinson's disease, they have had time to reflect on what is important in life.

Freddy is the eldest of the group interviewed. He does not just reflect on what his experience has taught him but also describes how his children's values have been affected by his PD journey.

It has taught them that no matter what happens in your life, you know there'll always be something which you'll have to get used to. You know it's not going to be the perfect life

This statement shows how Parkinson's and DBS can also affect the values of family members. Irrespective of his return to better health, Freddy remains grounded in the understanding that his physical state will most probably still deteriorate over time and hence he does not get overly excited by his improved state.

If I were to go today I'd, I'd feel quite happy with, with things. I don't long for anything, I don't look back.

Freddy has quite a stoic attitude. He remains grateful to live in the moment without regret. His more advanced age could explain his current pragmatism. He describes how PD and DBS motivated him to draw up his will and get other financial affairs in order. However, the younger members of the group interviewed put a great deal of emphasis on living life and maintaining their physical state, but for Freddy, getting affairs into order for his wife and children has become a priority. He now values stability and predictability and does his best to ensure his family's future.

#### 3.2.4. The Future Is Less Predictable

For many, DBS has not removed the fear of deterioration. Surgery can have an almost miraculous outcome at the start, but as time passes, some patients become more realistic about the trajectory of their disease.

Barry: *I feel more confident and then also at the same time we are still on a downhill slope so that confidence is still being taken away from me everyday*

As much as DBS interrupts the life course of PD, the eventual progressive nature of the disease means that the future remains uncertain. This knowledge was interpreted in different ways according to the participants but most had a long-term view of the future and a fear of going “backwards.”

Only a couple of men found the future a more reassuring place because, “*I know full well that I will be at this level in 10 years*,” states Dean.

Dean cannot know this for sure, but it would seem that DBS has given him hope and it is through this lens he chooses to see his future.

No PD patient can really know the trajectory of their illness; however, DBS can improve the outlook for the future. Like Dean, some were able to enjoy the present without focusing too much on the long-term future. However, most resonated with Barry's sentiment that the future is uncertain. DBS offers a hope that the future will be better with the implants than without. However, it is rare that all fear of deterioration is eradicated.

## 4. Discussion

What the results of this study suggest is that the merging of the self with PD that occurs as the disease progresses is moderated by DBS surgery. Post-DBS patients endeavour to work out who they are in the face of such great change as they are rarely, truly “back to normal.” This therefore demands new interpretations of social roles, perceptions of control, the nature of help, and views of the future. For all participants interviewed, life with DBS is an improvement on life before surgery. However, for most, there remains a sadness that DBS can never truly separate them from their Parkinson disease with which their identity seems to be merged. Most often, this loss of independent identity was only realized retrospectively. Many patients described a similar experience as previously reported, whereby the scars left by DBS were a mark of honour [10].

DBS signified the beginning of great change for all interviewed, but the duration of these positive changes varied, hence the first theme, “Healed and relieved: all that glitters is not gold.” The perioperative phase was a moment of true delight for all the patients' interviewed as the switching on of the stimulator became synonymous with a switching on of new hope. This was the moment most patients became aware of a return to themselves. This was illustrated by Henry's comment, “I just went back to the meaning of normal.” As noted in a previous study [14], it was only after the initial period of elation subsided that patients became aware of their postsurgical capabilities. For some, the eradication of their most troubling symptoms was enough to have them feel “normal;” for others, the awareness that certain abilities still remained diminished and that new issues had arisen after surgery was disappointing. This has been noted in most qualitative studies focusing on life after DBS surgery (for review, see [8]). Patients were left to reflect on the life they once lived and to compare it to their current reality. All accepted the good and bad that came with DBS as a fair trade for the presurgery life.

In the second theme, “The change within: new interpretations of the present and future unfold,” an effect of age emerged, which has not been explored in the existing qualitative literature that focuses on life after DBS surgery. It was the older participants such as Freddy who were able to take quite a stoic view of their PD at its worst, “*I think I'd achieved everything I wanted to achieve and so I did not feel bad about not going out, I did not feel punished or anything, I just thought oh this is the way it is you know*.” DBS was seen as a gift of time. There was an acceptance that PD would progress and the participants would age and therefore, life should be made the most of. There was less talk of needing to work on relationships or needing to do more. DBS was an addition to life that would just make life more bearable.

The patients with younger onset, such as Gary, predominantly saw their presurgery lives as dominated by their PD, “*I always said it's the kind of disease that would make me a hermit if I wanted to crawl up into corner and just stay there on my own*.” Even with the positive changes DBS delivered, they found it hard to view their life through any other lens than that of the damaged and declining PD patient. Barry stated, “*Obviously I still have PD so it obviously gets worse every day*.” For this subset of participants, the sense of the clock ticking felt slightly more urgent and it felt as if there was a stronger need to reinterpret one's life and one's place in the world. However, unlike in other studies [13, 15, 16], the participants interviewed in this study showed a more positive acceptance of DBS. Knowing that symptoms have a likelihood of returning over time, many patients worked on the relationships they had within their social support network. For some, this network was always valued; for others, PD and DBS was a good wake up call, making them realize how essential good support can be.

These patients were also left working out to what extent they still had control over their bodies, their social lives, and their future. These views were often murky and unclear. Barry succinctly puts this when he explains, “*I feel more confident and then also at the same time we are still on a downhill slope so that confidence is still being taken away from me every day*.” We see Barry's continuing struggle to take control of his life now that his PD symptoms have been alleviated. However, we also sense Barry's understanding that control may just be an illusion as his PD continues to progress in the background.

The introduction of a device with a remote control means that patients are physically handed power over some of their symptoms. The confusion and sometimes unwillingness to take this control is interesting. Patients have spent years surrendering control to their illness, carers, and physicians. For most, the idea of having control returned to them but through a device external to their body was confusing and a good illustration of how patients needed time to reinterpret the new changes they faced. Most needed a period of time to accept such a device and some had yet to fully accept it, preferring the comfort of maintaining their spouse in their caring role by handing them the remote control.

The most unifying result from this study was the overwhelming sentiment of gratitude. No one took DBS for granted. Whether they had been operated on 1 or 10 years ago, whether they had experienced great or moderate symptomatic benefit, all the patients were grateful for their surgery and would recommend it. Many felt indebted to the surgical team and society in general making them more compassionate and empathetic towards the struggles of others. This sense of gratitude even helped to shape the view of the future for some.

The unpredictability of the future was apparent in most interviews and again is an aspect reported in other studies [14]. These interviews showed an acknowledgement of the power of DBS and medical research. Even if there was fear of deterioration, most maintained a level of hope for the future. The future for all looked better with DBS than it did without.

## Figures and Tables

**Table 1:** Pseudonyms, age, duration of PD, and time since DBS surgery in years.

Name	Age (years)	Duration of PD (years)	Time since DBS (years)
Adam	63	17	2
Barry	46	17	3
Charles	52	11	1
Dean	60	12	10
Ed	53	13	3
Freddy	67	14	2
Gary	65	16	1
Henry	60	16	3
Ira	57	17	5
James	60	10	1

## Data Availability

Data are not available due to confidentiality.
